# Evaluating cepharanthine analogues as natural drugs against SARS‐CoV‐2

**DOI:** 10.1002/2211-5463.13337

**Published:** 2021-12-09

**Authors:** Atsushi Hijikata, Clara Shionyu‐Mitsuyama, Setsu Nakae, Masafumi Shionyu, Motonori Ota, Shigehiko Kanaya, Takatsugu Hirokawa, Shogo Nakajima, Koichi Watashi, Tsuyoshi Shirai

**Affiliations:** ^1^ Faculty of Bioscience Nagahama Institute of Bio‐Science and Technology Japan; ^2^ Department of Complex Systems Science Graduate School of Informatics Nagoya University Japan; ^3^ Computational Biology Laboratory Division of Information Science Graduate School of Science and Technology Nara Institute of Science and Technology (NAIST) Ikoma Japan; ^4^ Division of Biomedical Science Faculty of Medicine University of Tsukuba Japan; ^5^ Transborder Medical Research Center University of Tsukuba Japan; ^6^ Cellular and Molecular Biotechnology Research Institute National Institute of Advanced Industrial Science and Technology Tokyo Japan; ^7^ Department of Virology II National Institute of Infectious Diseases Shinjuku‐ku Japan; ^8^ Research Center for Drug and Vaccine Development National Institute of Infectious Diseases Shinjuku‐ku Japan; ^9^ Department of Applied Biological Sciences Tokyo University of Science Noda Japan

**Keywords:** coronavirus, drug repurposing, molecular docking, natural drug, SARS‐CoV

## Abstract

Cepharanthine (CEP) is a natural biscoclaurine alkaloid of plant origin and was recently demonstrated to have anti‐severe acute respiratory syndrome coronavirus 2 (anti‐SARS‐CoV‐2) activity. In this study, we evaluated whether natural analogues of CEP may act as potential anti‐coronavirus disease 2019 drugs. A total of 24 compounds resembling CEP were extracted from the KNApSAcK database, and their binding affinities to target proteins, including the spike protein and main protease of SARS‐CoV‐2, NPC1 and TPC2 in humans, were predicted via molecular docking simulations. Selected analogues were further evaluated by a cell‐based SARS‐CoV‐2 infection assay. In addition, the efficacies of CEP and its analogue tetrandrine were assessed. A comparison of the docking conformations of these compounds suggested that the diphenyl ester moiety of the molecules was a putative pharmacophore of the CEP analogues.

## Graphical abstract

Cepharanthine is a natural biscoclaurine alkaloid of plant origin and has recently been demonstrated to have anti‐severe acute respiratory syndrome coronavirus 2 activity. To evaluate the cepharanthine analogues as a potential coronavirus disease 2019 drug, we performed docking simulation and a cell‐based infection assay. From the results, the diphenyl ester moiety of the molecules was suggested for a putative pharmacophore of the cepharanthine analogues.

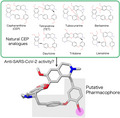

**Abbreviations**
ACE2: angiotensin‐converting enzyme 2
AD4: AutoDock 4
ADV: AutoDock Vina
CEP: cepharanthine
COVID‐19: coronavirus disease 2019
M‐pro: main protease
MproS: M‐pro pocket site
NPC1: Niemann–Pick type C intracellular cholesterol transporter 1
PC: principal component
PCA: principal component analysis
PDB: Protein Data Bank
RBD: receptor binding domain
RMSD: root‐mean‐square deviation
SARS‐CoV‐2: severe acute respiratory syndrome coronavirus 2
S‐pro: spike protein
SproS: S‐pro receptor binding domain
TET: tetrandrine
TPC2: two pore segment channel 2

Despite the successful developments and applications of the coronavirus disease 2019 (COVID‐19) vaccines [1, 2], considerable numbers of breakthrough infections are still threatening the world’s health [3, 4]. Also, the emergence of novel variants of severe acute respiratory syndrome coronavirus 2 (SARS‐CoV‐2), which escape from the immunity developed through the vaccinations, is highly concerning [5, 6]. These facts should underline the still‐ongoing requirement of effective anti‐SARS‐CoV‐2 therapeutics.

Some of the authors recently reported that a combined treatment of nelfinavir and cepharanthine (CEP) was highly effective for COVID‐19 by screening a panel of the approved drugs in a SARS‐CoV‐2 cell culture model [7]. Nelfinavir and CEP are the approved drugs for anti‐AIDS and anti‐leukopenia, respectively. CEP hinders SARS‐CoV‐2 entry into cells, and nelfinavir inhibited the catalytic activity of viral main protease (M‐pro) to suppress viral replication. *In vitro* assay confirmed a synergistic effect of the combined treatment to suppress SARS‐CoV‐2 proliferation. Nelfinavir was predicted to shorten the period before viral clearance by ~5 days by treatment at early days postinfection, and the additional treatment of CEP enhanced the efficacy significantly.

CEP is a natural product isolated from the plant *Stephania cephalantha Hayata* and has been approved for leukopenia, xerostomia and alopecia to date [8]. Natural products such as CEP are expected to be promising sources of anti‐COVID‐19 therapeutics [9, 10, 11, 12, 13]. It is well known that natural drugs exhibit antiviral activities against notable viral pathogens, including coronavirus, dengue virus, coxsackievirus, hepatitis B virus, hepatitis C virus and influenza virus [14, 15]. Therefore, so far, many studies for nature‐derived drug discovery have been conducted against SARS‐CoV‐2 by *in silico* virtual screenings [16, 17, 18], as well as *in vitro* assays [19, 20]. For instance, quercetin, which is a plant flavonoid and known to have antiviral properties, has been confirmed to block the proteolytic activity of SARS‐CoV‐2 3CLpro *in vitro* [19] and is currently in a phase 3 clinical trial (ClinicalTrials.gov identifier: NCT04578158). The other flavonoids, such as baicalein, herbacetin and pectolinarin, have also been discovered as potent inhibitors for the enzyme [20].

Thus, the efficacies of natural CEP analogues against COVID‐19 would be worth further investigating. In this study, an *in silico* docking study of natural CEP analogues and a cell‐based assay of anti‐SARS‐CoV‐2 activity for selected analogues have been attempted.

## Materials and methods

### Search for CEP analogues

The CEP analogues were sought in the KNApSAcK database [21]. The CEP structure was exhaustively compared with the compounds in the database by using COMPLIG [22]. COMPLIG matches molecular graphs and evaluates the similarity score of two molecules, A and B, as min{M(A, B)/M(A), M(A, B)/M(B)}, where M(A), M(B) and M(A, B) are the total numbers of atoms and bonds in molecules A and B, and the total number of atoms and bonds matched between molecules A and B, respectively. Both element and chirality should be identical for atoms, and bond order should be identical for bonds to be matched, if applicable. A total of 24 compounds with more than 0.90 similarity score were extracted (Table 1). COMPLIG was also used to superimpose the compound structures according to the graph matching.

**Table 1:** CEP analogues and M‐pro inhibitors

Compound name	KNApSAcK CID	COMPLIG score	Assay^a^	ADV best score					AD4 best score					RMSD between best docking poses^b^					
				SproS	NPC1S1	NPC1S2	TPC2S	MproS	SproS	NPC1S1	NPC1S2	TPC2S	MproS	SproS	NPC1S1	NPC1S2	TPC2S	MproS
CEP	C00001836	1.00	*	−6.8	−9.5	−9.3	−11.6	−7.9	−5.7	−9.2	−10.5	−9.0	−9.4	0.9	1.3	2.3	3.8	2.5
Daphnandrine	C00001843	0.99		−6.6	−8.8	−9.0	−11.7	−7.5	−5.7	−9.1	−10.1	−9.2	−9.4	3.6	2.3	2.5	4.0	3.2
Trilobamine	C00001844	0.97		−6.5	−9.1	−9.9	−11.4	−8.3	−5.7	−8.5	−9.4	−9.2	−8.7	3.4	2.4	0.8	4.1	1.2
Oxyacanthine	C00001897	0.97		−6.1	−9.0	−9.1	−11.0	−7.5	−5.1	−8.8	−9.7	−8.3	−8.3	3.8	2.9	2.5	2.7	2.7
Thalmine	C00001922	0.96		−7.0	−8.9	−8.6	−10.4	−8.6	−6.2	−9.9	−9.4	−8.5	−8.3	0.8	2.6	3.7	2.8	1.8
O‐Methylthalicberine	C00001888	0.96		−6.0	−9.1	−8.9	−9.5	−6.5	−5.2	−9.3	−8.9	−9.2	−7.8	3.8	1.9	2.6	3.3	2.9
Liensinine	C00028473	0.96	*	−6.9	−8.0	−8.5	−9.7	−7.9	−5.9	−8.8	−9.2	−7.1	−9.4	1.6	2.5	3.4	3.7	2.7
TET	C00001919	0.95	*	−6.6	−8.4	−9.3	−9.3	−7.1	−5.5	−9.6	−9.5	−9.1	−9.5	3.6	2.3	3.6	2.1	2.8
Tubocurarine	C00001927	0.95	*	−6.5	−9.4	−9.1	−11.6	−7.6	−6.0	−8.6	−10.1	−8.1	−9.3	4.0	1.8	3.6	3.3	2.7
Trilobine	C00001926	0.95	*	−7.1	−9.9	−9.9	−12.5	−8.9	−6.1	−9.6	−10.4	−8.9	−11.6	4.0	1.3	1.1	4.1	1.7
Isotrilobine	C00025914	0.95		−6.8	−9.4	−9.5	−12.6	−8.4	−6.1	−10.3	−10.2	−9.2	−10.7	2.2	3.0	2.4	3.5	2.6
Aromoline	C00001811	0.94		−6.6	−9.0	−10.1	−11.2	−7.7	−5.5	−8.8	−9.5	−8.0	−8.9	1.8	2.6	0.8	3.9	2.7
Pycnamine	C00001909	0.94		−6.4	−9.0	−10.0	−11.5	−7.1	−5.9	−8.8	−9.5	−8.9	−11.0	3.7	2.5	2.2	0.8	2.5
Dauricine	C00001845	0.94	*	−7.1	−8.0	−9.6	−9.8	−7.9	−5.6	−10.2	−10.5	−7.7	−8.7	2.8	3.1	3.0	3.7	2.7
Magnoline	C00025880	0.94		−6.8	−9.4	−10.6	−10.3	−9.0	−5.4	−11.2	−10.4	−7.8	−10.2	4.2	3.3	1.6	3.8	2.2
Hypoepistephanine	C00050801	0.94		−6.1	−9.6	−9.4	−10.9	−7.4	−5.3	−8.6	−8.2	−9.0	−8.4	3.9	2.6	2.2	1.6	2.5
Berbamine	C00001817	0.92	*	−6.4	−9.0	−10.0	−11.6	−7.2	−5.9	−8.8	−9.5	−9.0	−11.0	3.7	2.5	2.2	2.9	2.5
Curine	C00025602	0.92		−7.3	−9.5	−9.2	−10.4	−7.4	−5.6	−9.3	−10.2	−9.4	−8.8	4.1	2.8	1.2	2.9	2.5
Isochondrodendrine	C00001870	0.92		−6.6	−9.2	−8.6	−10.2	−7.2	−5.6	−8.7	−9.4	−7.7	−9.7	2.9	4.7	2.5	2.4	2.7
Bebeerine	C00001816	0.92		−6.5	−9.3	−8.9	−11.1	−7.4	−5.6	−10.3	−9.3	−8.3	−9.6	4.1	2.0	1.0	3.4	2.8
Kurramine‐2'‐beta‐*N*‐oxide	C00051175	0.92		−7.5	−10.1	−10.8	−12.7	−10.4	−6.3	−9.9	−10.8	−9.3	−11.3	2.4	4.6	0.9	2.4	0.9
Thalsimine	C00001923	0.91		−6.2	−8.4	−9.1	−9.6	−6.9	−5.8	−8.4	−8.4	−9.4	−8.8	2.5	2.0	3.3	3.2	2.3
Thalicarpine	C00001920	0.91		−6.9	−9.5	−9.0	−9.3	−8.4	−4.9	−8.9	−7.1	−8.6	−8.8	3.9	3.1	4.0	3.7	2.7
Kurramine‐2'‐alpha‐*N*‐oxide	C00027148	0.91		−7.6	−9.7	−10.1	−12.2	−11.3	−7.4	−9.5	−11.3	−9.3	−10.8	3.6	2.7	3.6	3.8	3.0
Hyperoside	C00005372	0.69	*	−7.2	−8.4	−8.1	−9.2	−9.3	−8.6	−10.8	−8.9	−9.2	−11.0	1.3	2.9	2.0	3.4	2.5
Baicalin	C00001024	0.66	*	−7.2	−8.8	−9.1	−9.9	−9.3	−7.9	−8.2	−8.9	−9.3	−10.6	3.5	2.8	2.8	4.1	2.4
Hesperidin	C00000970	0.65	*	−6.6	−9.8	−10.0	−10.9	−9.0	−8.1	−9.3	−9.3	−11.1	−10.9	2.8	1.6	1.6	2.8	3.0

^a^ The compounds used for the assay are indicated with an asterisk.

^b^ RMSD (Å) of the best‐scored ligand atoms between ADV and AD4.

### Docking study of CEP analogues and M‐pro inhibitors

The binding affinities of the CEP analogues and M‐pro inhibitors to the receptor binding domain of spike protein (S‐pro) and M‐pro of SARS‐CoV‐2, Niemann–Pick type C intracellular cholesterol transporter 1 (NPC1) and two pore segment channel 2 (TPC2) of human were predicted via molecular docking simulations. The target atom coordinates of S‐pro and M‐pro were adapted from the previously reported complex models [12] based on the Protein Data Bank (PDB) [23] codes 6M0J [24] and 6LU7 [9], respectively. The NPC1–NPC2 complex model was constructed based on PDB code 6W5V [25]. The TPC2 coordinates were based on PDB code 6NQ0 [26]. Note that the resolutions of the latter two structures were lower than the recommended threshold for a docking simulation, 2.5 Å, specifically, 4.00 and 3.70 Å for 6W5V and 6NQ0, respectively. The target coordinates in complex with the best‐scored CEP are available from https://harrier.nagahama‐i‐bio.ac.jp/dtx/SARS‐CoV‐2/.

In the previous study, knowledge‐based modeling, in which the similar ligands in the known complex structure were used to predict the interactions, was applied for modeling the drug–SARS‐CoV‐2 protein complexes [12]. However, no complex structure binding a CEP analogue was detected among the known homologous structures. Thus, a docking simulation was used in this study. autodock vina (ADV) version 1.1.2 was used to dock the model compounds to the proteins [27]. autodocktools version 1.5.6 was used to prepare PDBQT files of the target proteins and compounds [28]. The concaved areas on the protein surface (pockets) were detected using fpocket2 [29]. The pockets overlapping the angiotensin‐converting enzyme 2 (ACE2) interaction site of S‐pro and the active site of M‐pro were selected for the target site. For NPC1 and TPC2, preliminary docking simulations of CEP were executed, and two and one pockets, respectively, with the highest docking score were selected for the target sites. The centers of the docking grids (28 × 28 × 28 Å) were defined as the centroid coordinates of amino acid residues composing each pocket (Fig. S2). The size and center of grids were manually modified for S‐pro to eliminate the ligands docked outside the ACE2 interacting region (24 × 24 × 24 Å). The docking simulations by ADV were performed with the options exhaustiveness of 1024 and seed of 1024. To verify the results, we also executed the docking simulations using autodock 4.2.6 (AD4) [30] with the same grid setting (center and size) and PDBQT files as ADV docking. To set the docking grid size to 28 × 28 × 28 Å, the grid point spacing was changed from 0.375 to 0.2 Å. The AD4 docking simulations were executed using the Lamarckian genetic algorithm with default parameters.

The molecular graphics were prepared by using UCSF Chimera [31]. The schematic diagrams of the interactions in the binding sites were prepared by using MOE [32]. The principal component (PC) analysis (PCA) of the docking scores was executed by using the r package [33]. The matrix of the best docking scores of the compounds for S‐pro receptor binding domain (SproS), main protease pocket site (MproS), NPC1S1, NPC1S2 and TPC2S sites (Table 1) was applied to the prcomp function of R with scaling for the PCA. To examine the contribution of each target site/protein to PC axes, we calculated the factor loadings (vector of correlation coefficients between the docking score axes and the PC axes). The docking scores were sign inverted so that the factor vectors pointed to the higher‐affinity directions.

### SARS‐CoV‐2 infection assay

The selected compounds for the infection assay experiment were purchased from Adipogen Life Sciences (Liestal, Switzerland; hesperidin, Cat. No. 83388), Chem Scene LLC (Monmouth Junction, NJ, USA; baicalin, CS‐5302), Med Chemexpress Co., Ltd (San Diego, CA, USA; dauricine, HY‐N0220; berbamine, HY‐N0714), Nacalai Tesque Inc. (Kyoto, Japan; tubocurarine, 35637‐849), Sigma Aldrich (St. Louis, MO, USA; CEP, SMB00418; hyperoside, 83388), Tim Tec LLC (Newark, DE, USA; trilobine, HTS11338), Tokyo Chemical Industry Co., Ltd. (Tokyo, Japan; tetrandrine [TET], T3321) and Toronto Research Chemicals Inc. (Toronto, Canada; liensinine, L397833). The anti‐SARS‐CoV‐2 activities of the compounds were assayed in the cell culture model as reported previously [7]. In brief, VeroE6/TMPRSS2 cells (VeroE6 cells overexpressing transmembrane protease, serine 2 [34]) were inoculated with the SARS‐CoV‐2 Wk‐521 strain at a multiplicity of infection of 0.003 in Dulbecco’s modified Eagle’s medium (Fujifilm Wako Pure Chemical, Osaka, Japan) supplemented with 10% fetal bovine serum (Sigma Aldrich), 10 U·mL^−1^ penicillin, 10 mg·mL^−1^ streptomycin and 10 mm HEPES (pH 7.4) at 37 °C in 5% CO_2_ for 1 h, and unbound viruses were removed by washing. The compounds were treated in nine serial dilutions (30.00, 10.00, 3.30, 1.10, 0.36 and 0.12 µm, and three technical replicates were also performed for 5.00, 1.00 and 0.20 µm) for 24 h, and the amounts of extracellular viral RNA were measured. Viral RNA was extracted with a MagMAX Viral/Pathogen II Nucleic Acid Isolation kit (Thermo Fisher Scientific, Waltham, MA, USA) and quantified by real‐time RT‐PCR analysis with a one‐step quantitative RT‐PCR kit (THUNDERBIRD Probe One‐step qRT‐PCR kit; TOYOBO, Osaka, Japan) using the SARS‐CoV‐2‐specific primers and probe [35]. The relative viral RNA levels were calculated by setting that for DMSO control treatment as 1.0 and were plotted against the concentrations of each compound. The cell culture infection assay was handled in a biosafety level 3.

## Results

### CEP analogues

The natural products highly similar to CEP were extracted from the comprehensive species–metabolite relationship database KNApSAcK (Table 1). A total of 24 CEP analogue compounds showed more than 90% identity in atoms/bonds to CEP by the graph‐matching of chemical formula. CEP consists of two coclaurine moieties connected in a cycle. Accordingly, most of the detected analogues were biscoclaurine alkaloids of plant origins. Although the detected analogues largely complied with this molecular architecture of CEP, no one perfectly shared the stereochemistry at two chiral centers and the coclaurine moieties conjugating atom positions with CEP (Fig. S1). It suggested that CEP is a stereochemically unique molecule among the analogues.

Several target proteins have been proposed for the CEP analogues, such as the S‐pro and M‐pro of coronaviruses and NPC1 and TPC2 of human. However, identities of the target proteins have not been fully confirmed in most of the cases.

CEP is thought to interfere with the ACE2–S‐pro interaction, which is essential for SARS‐CoV‐2 entry into cells [24], because CEP inhibits the entry phase in viral infection [7]. Also, the interaction of CEP to NPC1 and its efficacy in NPC1 inhibition have been reported [36, 37, 38]. NPC1 is the cholesterol transporter, which acts in salvaging cholesterol from the endosomal or lysosomal compartment to the cell membrane. A dysfunction of NPC1 activity results in Niemann–Pick type C disease partly because of a disruption of membrane raft formation, which also works as an obstacle to viral entry into cells [39, 40, 41].

TET has been shown to have anti‐SARS‐CoV‐2 activity through inhibiting TPC2 [42]. TPC2 is an endosomal cation channel that acts in trafficking low‐density lipoprotein molecules and also is known to be involved in viral entry into cells through the endosome [43].

M‐pro is the most used drug target of SARS‐CoV‐2, and a few of the CEP analogues, including curine, are assumed to interact with this enzyme [44]. Therefore, for a comparison purpose, three natural products, which are thought to target M‐pro, have also been included in this analysis, namely, baicalein [20], hyperoside [45] and hesperidin [46].

### Docking study

**Fig. 1:**
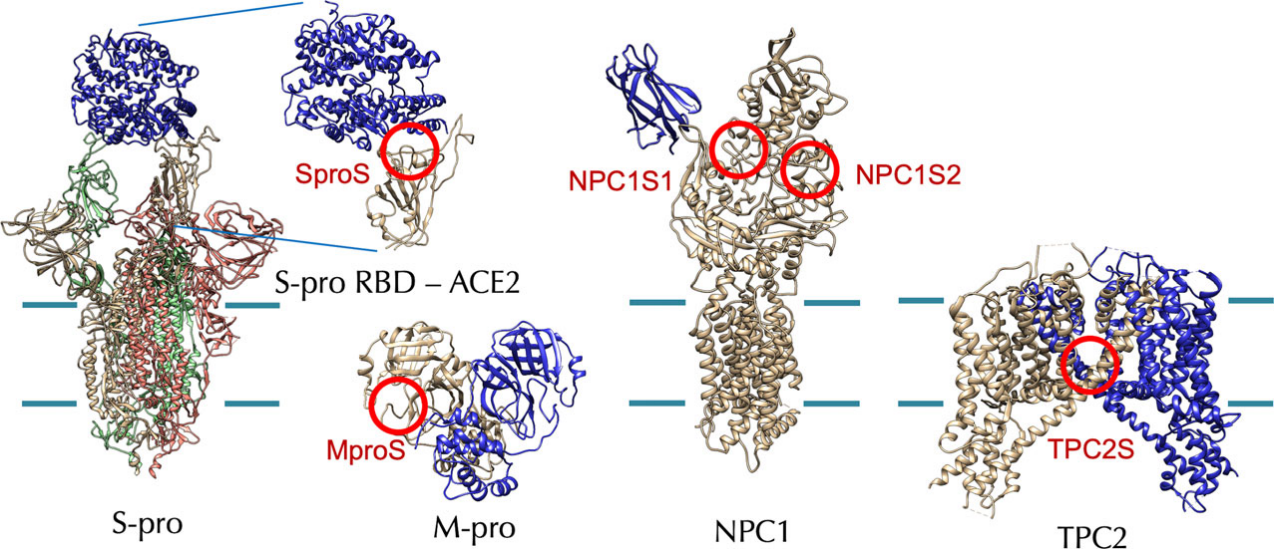
Hypothetical target proteins and target sites of CEP analogues. The target sites are indicated with red circles. The cell membrane boundaries are shown with green lines for the membrane proteins. RBD, receptor binding domain.

ACE2 interaction site of SproS [24], active site of M‐pro (MproS) [9], two putative CEP binding sites of NPC1 (NPC1S1 and NPC1S2) [25, 47] and the putative CEP binding site of TPC2 (TPC2S) [26] were targeted in this study (Figs 1 and S2).

Docking simulations of the CEP analogues and the M‐pro inhibitors to the hypothetical target sites were performed by using ADV [27] and AD4 [30], and the best‐scored docking poses for each pair of compound and target site were compared (Table 1). The score correlation between ADV and AD4 was not high, specifically, 0.48, 0.05, 0.49, 0.17 and 0.55 for SproS, NPC1S1, NPC1S2, TPC2S and MproS, respectively. It has been proposed that the docking poses were generally reliable if root‐mean‐square deviation (RMSD) of the ligand atoms between the ADV and AD4 were less than 2.0 Å [48]. The RMSD values tended to be lower for the suggested targets, for example, 0.9 and 1.3 Å for CEP against SproS and NPC1S1, respectively, and 2.1 Å for TET against TPC2S.

**Fig. 2:**
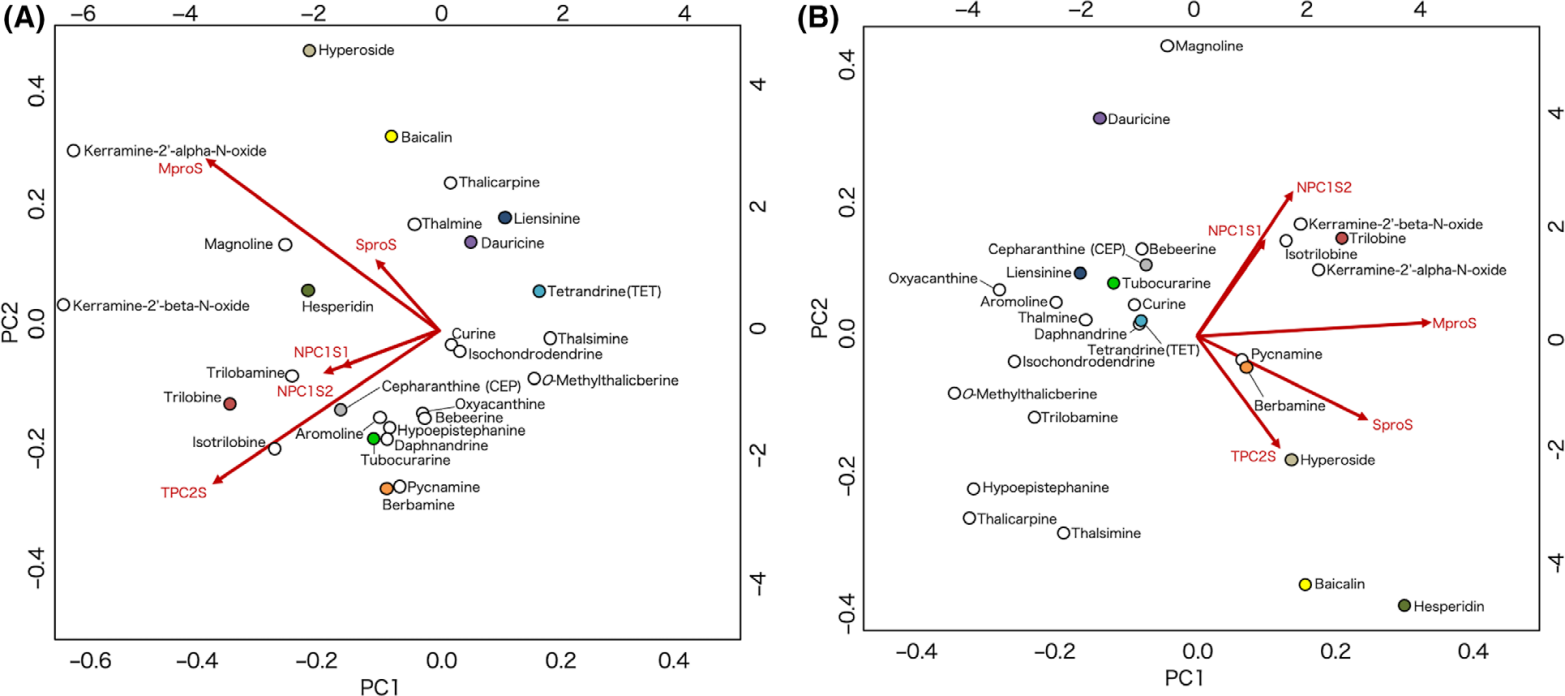
Compound distribution in PC1–PC2 plane of docking score. The distributions of the best‐scored compounds on PC1–PC2 planes are shown for (A) ADV and (B) AD4. The compounds are indicted with circles, where those used for assay are differently colored. The scales on the bottom and left of the plot are the PC scores of the compounds for the PC1 and PC2 axes, respectively. The loadings of sign‐inverted docking scores for the target sites to the principal axes are indicated as overlaid red arrows, and the scales on the top and right indicate the corresponding factor loadings. The figure was prepared by using biplot function of R of default settings and scaling.

To systematically analyze the results of docking simulation, we performed a PCA over the matrix of docking scores (Fig. 2). The cumulative proportion of variance was 86.5% to the second PC axis (59.1% and 27.4% for PC1 and PC2, respectively) for the ADV results. For the AD4 results, the cumulative proportion of variance was 71.2% to the second PC axis (48.5% and 23.5% for PC1 and PC2, respectively).

In the PC1–PC2 planes, the distributions of the compounds were different between ADV and AD4. As a consensus feature, the distributions were determined mainly by separating the viral proteins (S‐pro and M‐pro) and human proteins (NPC1 and TPC2). The M‐pro inhibitors (hyperoside, baicalin and hesperidin) preferred M‐pro, which was consistent with the hypothetical target of the compounds. CEP relatively preferred NPC1 but was placed closer to the center of the distribution. The other compounds also, as overall, did not show a bias to a specific target. A total of 10 procurable compounds were selected so that they surround CEP in the PC1–PC2 plane as indicated with colored circles in Fig. 2.

### SARS‐CoV‐2 infection assay

**Fig. 3:**
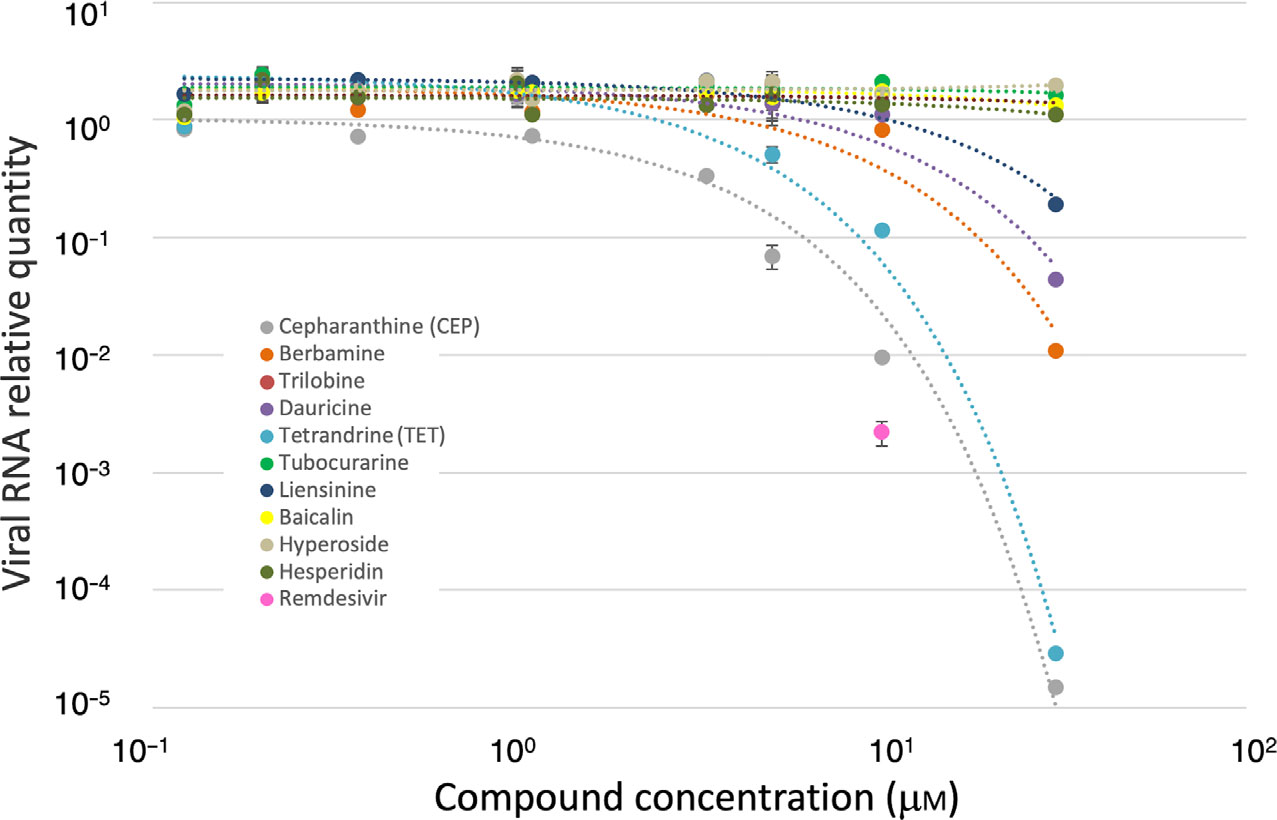
Dose–response curves of anti‐SARS‐CoV‐2 activity of compounds. The relative quantities of secreted viral RNA at 24 h after inoculation (vertical axis in log scale) for all technical replicates are plotted together against compound concentrations (horizontal axis in log scale). The colors for compound plots are coordinated with Fig. 2. The plot in magenta indicates the efficacy of remdesivir at a 10 µm concentration as a positive control. The error bars for 5.00, 1.00 and 0.20 µm plots indicate SDs (*n* = 3).

A cell culture SARS‐CoV‐2 infection assay was performed for the selected compounds. VeroE6/TMPRSS2 cells inoculated with SARS‐CoV‐2 for 1 h and washed out were incubated with or without the compounds for 24 h, and viral RNA in the culture supernatant was quantified (Fig. 3). As a result, CEP was most effective in suppressing viral proliferation (half maximal [50%] inhibitory concentration [IC_50_] and 90% inhibitory concentration [IC_90_] values of 1.90 and 4.46 µm, respectively), which was followed by TET (IC_50_ and IC_90_ values of 10.37 and 14.80 µm, respectively). Berbamine, dauricine and liensinine had even weaker activity to inhibit SARS‐CoV‐2. Other compounds did not show a significant effect. As a positive control, remdesivir is currently an approved anti‐COVID‐19 drug directly targeting a SARS‐CoV‐2 protein with IC_50_ value of 0.99 µm [49].

## Discussion

The natural CEP analogues have been examined for anti‐SARS‐CoV‐2 activity by combining *in silico* and *in vitro* analyses. The results indicated that CEP showed a potent activity to inhibit SARS‐CoV‐2 infection among the naturally occurring analogues, which confirmed the previous report [7]. TET was also shown to be effective but had lower activity than CEP. These two compounds are hypothesized to interact with human lysosomal membrane proteins: CEP to NPC1 and TET to TPC2. The natural ligands of these membrane proteins are cholesterol for NPC1 and phosphatidylinositol 3, 5‐bisphosphate for TPC2, and consequently the molecular environments of target sites are hydrophobic [25, 42]. The CEP analogues are generally hydrophobic molecules with predicted logP values ranging from 5.8 to 6.7, and those for CEP and TET are 6.5 and 6.4, respectively. Usually, discriminating specificity or evaluating affinity for a highly hydrophobic interaction is difficult. Accordingly, CEP and TET did not localize in the PC1–PC2 plane of the docking scores (Fig. 2), indicating no preference for a particular target was predicted. Thus, the result did not provide additional implications for the target protein of these compounds. CEP and TET are the natural products, and they have neither been designed nor tuned for a specific target protein. It would be probable that they weakly interact with multiple targets, for example, NPC1 and TPC2.

**Fig. 4:**
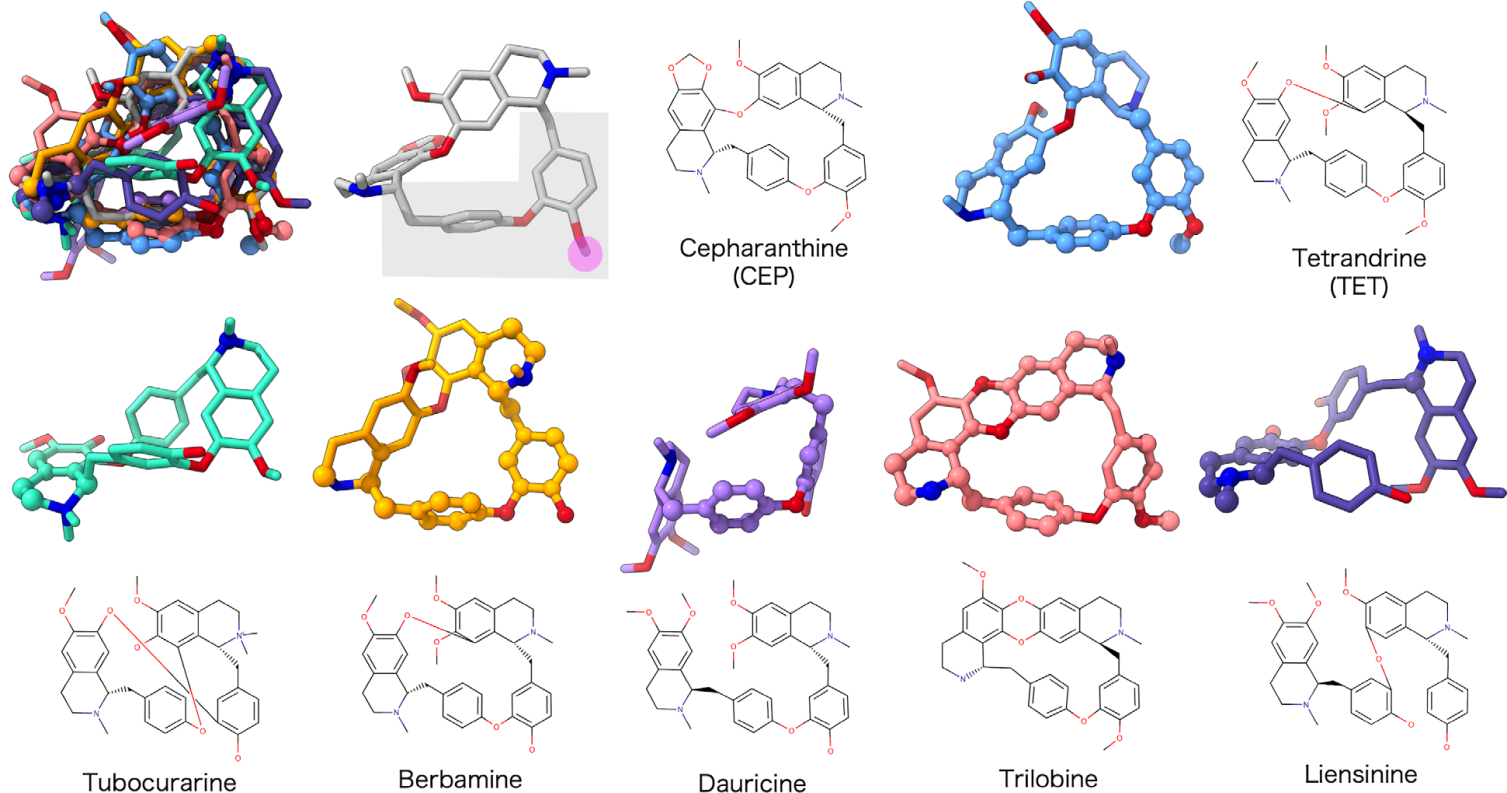
Comparison of effective and noneffective CEP analogues. 3D structures of the CEP analogues in the best‐docking‐scored conformations were superimposed to that of CEP in the upper left, which is followed by the separate depictions of the same structures. The colors for the models are coordinated with Fig. 2. The atoms within 1.7 Å from the graph‐matched atoms of CEP are shown in ball models. The suggested pharmacophore and its methyl moiety are meshed in gray and magenta, respectively.

Like many of the CEP analogues, CEP and TET are the cyclic molecules with conjugated coclaurine moieties, and their chemical formulas are highly similar. However, because of the difference in configuration at two chiral centers and the positions of the atoms for coclaurine moieties conjugation, the 3D structures of CEP and TET considerably deviated. Therefore, the 3D structures of the best‐docking‐scored CEP and TET and other examined analogues are superimposed to extract common structural features (Fig. 4). The overall structures were not superimposable between the analogues, including between CEP and TET. Most of the superimposable partial structures to CEP were centered by a diphenyl ester moiety for TET, berbamine, dauricine and trilobine as indicated with the ball‐and‐stick models in Fig. 4. Between CEP and TET, the conformations of the diphenyl ester moiety (meshed in gray for CEP in Fig. 4) were similar, and none of the other examined CEP analogues without remarkable anti‐SARS‐CoV‐2 activity had this CEP–TET structural motif in an identical manor. For example, berbamine and dauricine took a similar conformation at the corresponding parts but lacked the methyl group (meshed in magenta for CEP in Fig. 4), which was shared between CEP and TET. Trilobine had this methyl moiety, but the conformation of the diphenyl ester moiety was slightly deviated from CEP and TET. The result suggested this common partial structure between CEP and TET as a putative pharmacophore of CEP analogues.

For the CEP analogues to be used as anti‐COVID‐19 therapeutics, their mechanisms of action should be carefully investigated. It has been recently pointed out that the cationic amphiphilic compounds, including CEP, frequently cause cellular phospholipidosis, which is a lysosomal storage disorder characterized by the excessive accumulation of phospholipids. The extents of phospholipidosis were highly correlated with the antiviral efficacies of the compounds. Such compounds also included hydroxychloroquine, which was initially expected to be an anti‐COVID‐19 drug, but the clinical trials were, in some cases, terminated partly because of adverse effects [50].

Considering the assumed action mechanisms of CEP and TET, i.e., inhibition of endosomal lipid/steroid trafficking proteins, the same adverse effect would be difficult to avoid. The drug‐induced phospholipidosis is a frequently encountered hindrance in drug development, and recently it becomes clearer that ammonium cation groups in the molecules play a critical role in the symptom [51]. The structural motif suggested for CEP and TET does not contain, although exists close to, the ammonium cations of the molecules; thus, it might be useful in designing CEP derivatives with low adverse effect, for example, replacing ammonium cation without largely affecting the antiviral efficacies. In the docking poses, where higher similarity was observed between the ADV and AD4 results, the suggested pharmacophore was used for interactions with proteins, but the ammonium cations did not make remarkable bonds in all of the cases (Fig. S2B). It might also contribute to further improving CEP or TET to obtain more target‐specific (to reduce cross‐reaction) or less specific (to be multitargeted) drugs.

## Conclusion

The natural CEP analogues were examined for anti‐SARS‐CoV‐2 activity, and the efficacies of CEP (IC_50_ 1.90 µm) and TET (IC_50_ 10.37 µm) were detected. The diphenyl ester moiety of the molecules was suggested for a putative pharmacophore, but no target protein preference was implied by the present analyses; therefore, it remained a possibility that they were weak multitargeted natural drugs.

## Conflict of interest

The authors declare no conflict of interest.

## Author contributions

MO, SK and TS conceived and designed the study. AH, CS‐M, SN, MS, TH and TS executed the modeling and docking studies. SN and KW did the infection assay. AH, CS‐M, SN, MS and TS wrote the manuscript. All authors commented on the manuscript.

## Supporting information


**Fig S1.** Chemical formulas of CEP‐analogues and M‐pro inhibitors. The formulas are arranged to be comparable with that of CEP except for the M‐pro inhibitors. Two chiral centers and connecting carbon atoms of coclaurine moieties of CEP are indicated with blue and green circles, respectively. The compound names are shown in red (CEP), blue (assayed CEP‐analogues), green (M‐pro inhibitors), or black (others).
**Fig S2.** Detail of docking sites on target proteins. (A) The closeup views of the target sites are shown for SproS, MproS, NPC1S1, NPC1S2, and TPC2S. The boxes depicted the search area for the ligands. The best score poses of CEP are shown in ball and stick models for each target site. (B) Schematic diagrams of the interactions of TET and CEP in the target sites (SproS, NPC1S1, and TPC2S), where the best score poses showed lower RMSD (≤ 2.1 Å) between ADV and AD4 results. The keys for the interactions were shown under diagrams. The suggested pharmacophore and ammonium cations are meshed in gray and red, respectively.

## Data Availability

The target coordinates in complex with the best‐scored CEP are available from https://harrier.nagahama‐i‐bio.ac.jp/dtx/SARS‐CoV‐2/.
