# Supplementary material for: Evaluating cepharanthine analogues as natural drugs against SARS‐CoV‐2
Source: FEBS Open Bio. 2021 Dec 9;12(1):285–94. doi: 10.1002/2211-5463.13337 (PMC8727928; doi:10.1002/2211-5463.13337)
Supplement: Supplementary file 1 — Fig S1. Chemical formulas of CEP‐analogues and M‐pro inhibitors. The formulas are arranged to be comparable with that of CEP except for the M‐pro inhibitors. Two chiral centers and connecting carbon atoms of coclaurine moieties of CEP are indicated with blue and green circles, respectively. The compound names are shown in red (CEP), blue (assayed CEP‐analogues), green (M‐pro inhibitors), or black (others). Fig S2. Detail of docking sites on target proteins. (A) The closeup views of the target sites are shown for SproS, MproS, NPC1S1, NPC1S2, and TPC2S. The boxes depicted the search area for the ligands. The best score poses of CEP are shown in ball and stick models for each target site. (B) Schematic diagrams of the interactions of TET and CEP in the target sites (SproS, NPC1S1, and TPC2S), where the best score poses showed lower RMSD (≤ 2.1 Å) between ADV and AD4 results. The keys for the interactions were shown under diagrams. The suggested pharmacophore and ammonium cations are meshed in gray and red, respectively. [file FEB4-12-285-s001.pdf]

Supplementary Data

Evaluating cepharanthine analogues as natural drugs against SARS-CoV-2

Atsushi Hijikata, Clara Shionyu-Mitsuyama, Setsu Nakae, Masafumi Shionyu, Motonori Ota, Shigehiko Kanaya, Takatsugu Hirokawa, Shogo Nakajima, Koichi Watashi, Tsuyoshi Shirai

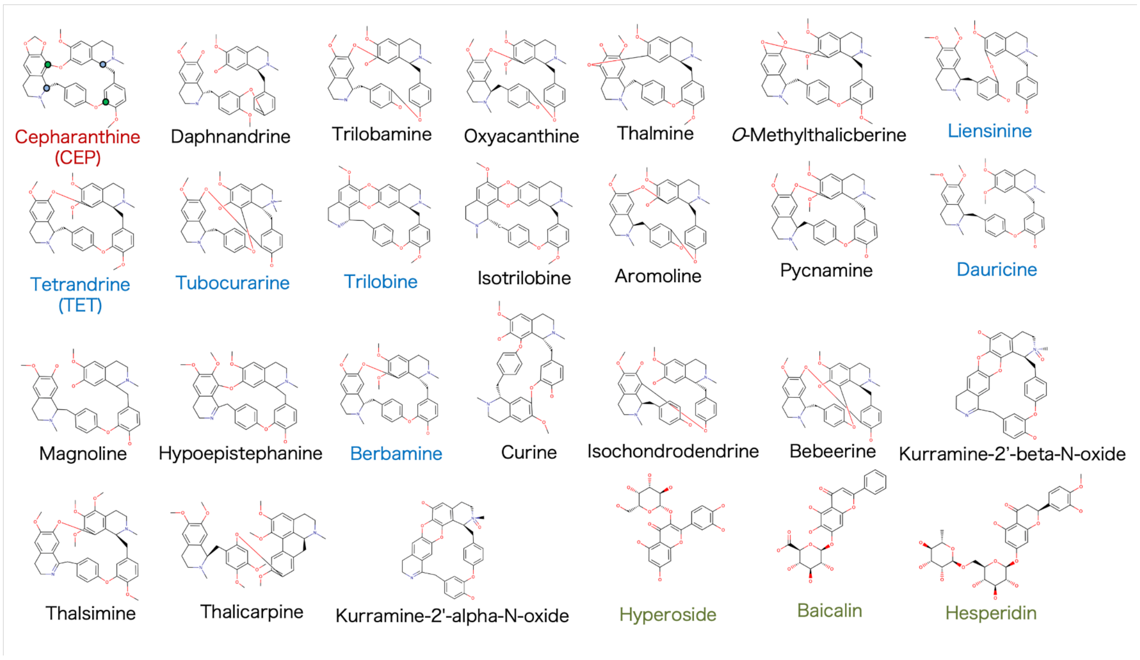

Fig. S1 Chemical formulas of CEP-analogues and M-pro inhibitors

The formulas are arranged to be comparable with that of CEP except for the M-pro inhibitors. Two chiral centers and connecting carbon atoms of coclaurine moieties of CEP are indicated with blue and green circles, respectively. The compound names are shown in red (CEP), blue (assayed CEP-analogues), green (M-pro inhibitors), or black (others).

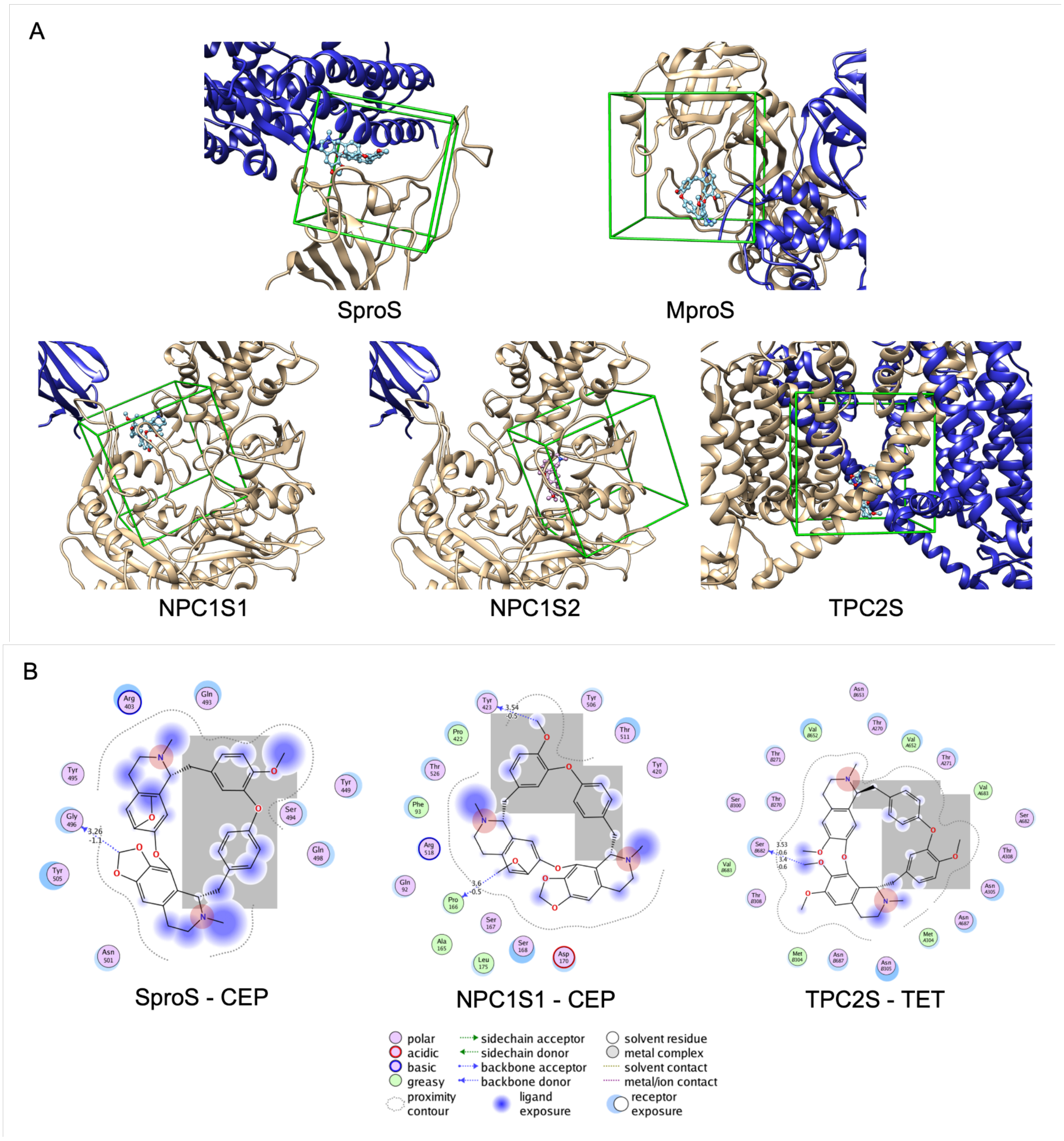

Fig. S2 Detail of docking sites on target proteins

(A) The closeup views of the target sites are shown for SproS, MproS, NPC1S1, NPC1S2, and TPC2S. The boxes depicted the search area for the ligands. The best score poses of CEP are shown in ball and stick models for each target site. (B) Schematic diagrams of the interactions of TET and CEP in the target sites (SproS, NPC1S1, and TPC2S), where the best score poses showed lower RMSD (≤ 2.1 Å) between ADV and AD4 results. The keys for the interactions were shown under diagrams. The suggested pharmacophore and ammonium cations are meshed in gray and red, respectively.
